# Preliminary analysis of validation evidence for two new scales assessing teachers’ confidence and worries related to delivering mental health content in the classroom

**DOI:** 10.1186/s40359-019-0307-y

**Published:** 2019-06-10

**Authors:** Brooke Linden, Heather Stuart

**Affiliations:** Department of Public Health Sciences, Queen’s University, 21 Arch Street, Kingston, Ontario Canada

**Keywords:** Mental health, Teacher confidence, Education, Scale development

## Abstract

**Background:**

While mental health challenges in the classroom have increased over the past several years, existing research suggests that many educators feel unprepared to broach the topics of mental health and mental illness with their students. This paper outlines the development and gathering of preliminary evidence of validity for two new scales designed to assess teachers’ confidence and worries related to delivering mental health content in the classroom.

**Methods:**

Content evidence was collected through the use of two methods: a focus group held with members of the Elementary Teachers’ Federation of Ontario, and a consensus survey conducted among a sample of educational experts recruited from an Ontario university. Internal structure evidence was derived from the initial intake survey of an evaluation of a new online guide designed to give elementary school teachers the tools and knowledge to develop lesson plans related to mental health. Internal consistency reliability of test scores was estimated with Cronbach’s alpha.

**Results:**

Both scales loaded on a single dimension with all items loading strongly (factor loadings greater than .60). Cronbach’s alpha coefficients of .96 for scores on the Teacher Confidence Scale and .93 for scores on the What Worries Me Scale estimated strong internal consistency reliability.

**Conclusions:**

We identified two unidimensional scales measuring concerns educators may have about discussing the topic of mental health in a classroom setting. The Teacher Confidence Scale for Delivering Mental Health Content contains 12 items measuring educators’ confidence in delivering mental health related materials in the classroom. The What Worries Me Scale contains 11 items. These scales may be useful for evaluating programs, educational workshops, and other initiatives aimed at improving teachers’ abilities to provide mental health content in the classroom.

## Background

Mental health is marked by the dynamic ability to recognize, express, and modulate changes in one’s own emotions, empathize with others, and cope with the normal stresses of life [1]. Mentally healthy individuals can work productively and fruitfully, and are able to make contributions to their community [2]. Conversely, individuals who have a mental illness often experience reduced ability to function and cope effectively [3]. Among the most affected are youth, with the first onset of many mental illnesses occurring during childhood or early adolescence [4].

Epidemiologic studies have shown that the prevalence of mental health problems during adolescence is high [5]. In any given year, approximately one in five adolescents will experience significant psychosocial impairment due to a mental illness [2, 4], which translates into roughly one in five students in the average classroom [6]. Many more will experience psychosocial problems that have the potential to interfere with their daily functioning [3, 4, 6, 7]. In addition to impacting students’ emotional well-being, mental illnesses may impact academic achievement, with related outcomes including difficulty concentrating, lower grades, reduced engagement, negative attitudes about school, suspensions, and expulsions [7–10]. Perhaps most concerning is that adolescents who struggle with untreated mental health problems are significantly less likely than their peers to graduate from high school or to enrol in post-secondary education [8, 11]. Research suggests that the schools that are most successful in promoting students’ academic achievements are those that integrate students’ academic, social, and emotional learning [12].

The 2014 Ontario Child Health Study revealed that 11% of the 31,000 student respondents reported needing help for mental health problems, but less than half would be willing to ask for help at school [13]. Importantly, thinking people at school would not be able to help, and not knowing who to approach were among the most frequently identified reasons to not seek help in the school setting [13].

As prominent adult role models in students’ lives, teachers can play a major role in helping youth to navigate and respond to changes in their mental health. However, existing research suggests that many educators feel unprepared to broach the topics of mental health and mental illness with their students. While teachers have frequently reported witnessing mental health issues impacting student performance, they have also identified a number of barriers to promoting student mental health in the school setting [7]. Key among these is teachers’ lack of adequate training in dealing with children’s emotional health and well-being. In one study, only 4% of teachers strongly agreed that they had an adequate level of knowledge required to meet their students’ mental health needs [14]. In another, teachers expressed great interest in the mental health of their students, but almost all reported having received little to no child mental health training [15].

In partnership with the Mental Health Commission of Canada, a 2012 survey conducted by the Canadian Teachers’ Federation among nearly 4000 teachers across Canada revealed that over half (54%) agreed that “addressing mental illness is not considered a role/priority of the school,” with 24% strongly agreeing with this statement [7, 13]. Virtually all teachers (96%) reported an important need for additional knowledge and skills training in strategies for working with children who experience mental health-related challenges [7]. Similarly, in a survey conducted by the School Based Mental Health and Substance Abuse Consortium for the Mental Health Commission of Canada, teachers identified the need for additional professional development as one of the biggest challenges to implementing mental health programs and services in their schools [16].

This paper reports on the development of two new instruments designed to evaluate the effectiveness of an online teacher training guide for improving elementary school teachers’ (Grade 7 and 8) confidence in delivering mental health-related content in the classroom. In this study, we defined teachers’ confidence as belief in their ability to positively influence student learning about mental health and mental illness [17]. Rooted in Bandura’s social cognitive theory (1997), the construct of teachers’ self-efficacy, or confidence, has gone through a substantial evolution over the past several decades. Both Tschannen-Moran and Hoy [18] and Klassen and colleagues [17] have conducted detailed reviews of the evolution of instruments designed to evaluate teachers’ self-efficacy, critiquing existing scales for evaluating general judgements about one’s ability to teach, rather than investigating teachers’ confidence in their ability to teach in specific subject areas. Klassen and colleagues emphasized the importance of developing “domain-specific measures” to complement existing tools designed to assess teachers’ self-efficacy more generally [17]. Therefore, we sought to create a domain-specific measure to evaluate teachers’ confidence in their ability to deliver mental health-related content in the classroom after a review of the literature revealed no such scale in existence.

During the initial development and field testing of the confidence instrument, a second underlying construct of interest related to, but separate from, teachers’ confidence became evident: teachers attributed their lack of confidence to worrying about the unpredictability of bringing discussions about mental health into the classroom, and the potential negative outcomes. This led to the development of a second instrument assessing teachers’ worries. In this study, we defined worry as feelings of anxiety surrounding the potential negative outcomes related to teaching students about mental health. Guskey made a similar observation in reviewing contextual variables that affect teachers’ self-efficacy, noting that teachers were more confident in their ability to influence positive student outcomes than to prevent negative ones [19]. Therefore, we concluded that the development of this second instrument evaluating teachers’ worries would provide a more holistic view of teachers’ confidence, hypothesizing that teachers who scored higher on the worries scale (e.g., indicating more worry) would score lower on the confidence scale (e.g., indicating a lower level of confidence).

This paper reports on the processes used to create both of these instruments and gather preliminary validation evidence. More specifically, we outline: (a) the processes by which item pool development took place for each instrument, and (b) the collection and analysis of content and internal structure evidence for validity.

## Methods

The scales that are described in this paper were developed iteratively, through a series of steps. Following item pool development, we used a number of methods to analyze the content and internal structure evidence for each scale. Analyses were completed using SPSS, Version 24 and R, Version 3.4.1. This research received ethics clearance from Queen’s University’s Health Sciences and Affiliated Teaching Hospitals Research Ethics Board.

### Item Pool development

Items for the Teacher Confidence Scale for Delivering Mental Health Content (TCS-MH) were developed by: a) adapting items from the Tschannen-Moran and Hoy’s (2001) Teachers’ Sense of Efficacy Scale (TSES) and b) developing items based on expert opinion [20]. Table 1 details the TSES items that were reworded, and in some cases combined, to develop items that more specifically aligned with the topic area of mental health, reflecting teachers’ confidence in their ability to deliver this type of material in the classroom. A total of twelve items were developed for the TCS-MH using this strategy. An additional four items were developed based on expert opinion from educational experts with whom the authors had previously worked, creating a total of sixteen items on the initial TCS-MH. The resulting scale was scored using a 10-point Likert scale response option ranging from ‘not confident at all’ to ‘very confident’. Lower scores indicated lower confidence.

**Table 1:** Development of the Teacher Confidence Scale for Delivering Mental Health Content

Item pool development		Response process evidence	Order
Select TSES Items	Original TCS-MH Items	Reworded TCS-MH Items	
How much can you do to motivate students who show low interest in schoolwork?	1. I can motivate students to learn about mental health and illness	1. **I can spark interest** in learning about mental health	4
How much can you do to help your students think critically?	2. I can help my students to think critically about mental health and illness	2. Help my students to be **more aware of their** mental health	5
n/a	3. I can teach students how to find reliable information about mental illness	3. Teach students how to find reliable information about mental **health**	11
How much can you do to improve the understanding of a student who is failing?	4. I can help make students’ perceptions about mental illness more positive	4. **Help to break down stereotypes about mental health**	13
To what extent can you make your expectation clear about student behavior?	5. I can help students to become more aware of misconceptions related to mental illness and their negative impact	5. Help students **to learn about the negative impact of stigma.**	14
n/a	6. I can improve students’ knowledge of resources available to support their mental health	6.	15
How much can you do to adjust your lessons to the proper level for individual students? How well can you implement alternative strategies in your classroom? How well can you provide appropriate challenges for very capable students?	7. I can adjust my mental health lessons to meet the learning needs of different students	7. **Create engaging** mental health lessons **for my students.**	6
n/a	8. I can improve students’ ability to seek help for mental health difficulties	8.	16
n/a	9. I can improve students’ knowledge about mental health and illness	9. Improve students’ **general** knowledge about **mental health**	7
To what extent can you craft good questions for your students?	10. I can craft thoughtful questions about mental illness for my students to consider	10. Ask **my students engaging** questions about **mental health**	8
How well can you respond to difficult questions from your students?	11. I can answer general questions about mental health and illness that my students might have	11. Answer **my students’** general questions about **mental health**	1
How well can you establish routines to keep activities running smoothly?	12. I can establish activities and classroom content to reinforce students’ knowledge of mental health and illness	12. **Create classroom activities** that reinforce students’ knowledge of **mental health**	12
How much can you do to get students to believe they can do well in schoolwork? How much can you do to help your students value learning?	13. I can help students to learn to value their mental health	13.	9
How much can you do to control disruptive behavior in the classroom? How well can you keep a few problem students from ruining an entire lesson?	14. I can use students’ attitudes toward mental health and illness to create teachable moments	14. Use students’ attitudes toward **mental health** to create **learning opportunities**	10
To what extent can you make your expectations clear about student behavior?	15. I can create a mentally healthy classroom	15.	2
How much can you do to get through to the most difficult students?	16. I can advocate for the importance of learning about mental health and illness	16. Advocate for the important of learning about **mental health**	3

Note. Bolded text indicates wording changes that were made as a result of focus group testing

n/a indicates no TSES items were used to develop item

-- indicates no wording changes were suggested

The initial version of the TCS-MH was used in the 2016 pilot evaluation of the aforementioned online guide for improving teachers’ confidence in delivering mental health-related content in the classroom. Though one goal of this evaluation was to collect evidence in support of the TCS-MH’s validity, a lower than expected participation rate precluded formal psychometric analyses. However, one open-ended question included in the evaluation invited respondents to share whether anything continued to concern them regarding teaching their students about mental health-related topics. Teachers’ response to this question revealed a substantial amount of worry regarding the unpredictability of bringing discussions about mental health into the classroom setting. Because many of these worries were not captured by the TCS-MH, we developed a second What Worries Me Scale (WWMS) using these qualitative responses as an initial pool of items (Table 2). A total of ten items were developed for the WWMS by rewording, and in some cases combining, these qualitative responses. An additional six items were developed based on expert opinion, creating a total of sixteen items on the initial WWMS. The resulting scale was scored using a 10-point Likert scale response option ranging from ‘strongly disagree’ to ‘strongly agree’, with lower scores indicating lower levels of worry.

**Table 2:** Development of the What Worries Me Scale

Item pool development		Response process evidence
Original Qualitative Responses	Original WWMS Items	Reworded WWMS Items
“Saying the wrong thing that may trigger a student with an undiagnosed issue”	1. I worry I may trigger an emotional reaction in a student with a mental health difficulty	**–**
“I worry that speaking about mental health problems may cause some students to identify with mental health conditions that they truly do not possess”	2. Cause a student to identify with a mental illness that they do not have	–
“I am not qualified and am worried I will do more damage than good” “I don’t want to say or do something that makes things worse”	3. Do more damage than good	**–**
“Actually, sparking the idea into a student who is doing just fine and then them second guessing themselves”	4. Cause students to second-guess their own mental health.	**–**
“I worry about the glamorization of mental illness and the stigma”	5. Glamorize mental illness	**–**
“Being aware of the potential sensitivities of students with mental health problems and how to present in a way that does not make them feel that the class is focused on them”	6. Embarrass students who do have mental health difficulty	**Single out** a student who does have a mental health difficulty
“I don’t want to say something that will make things worse for a student who is struggling”	7. Make things worse for a student who has a mental health difficulty	**–**
“I am most worried about sharing incorrect information or information that is not appropriate for the students” “I am concerned that I do not have enough training to teach about mental health”	8. Convey inaccurate information	^a^
“I am worried about saying the wrong thing to the students” “I am worried I’ll say something that offends someone dealing with a mental health issue”	9. Offend someone that is dealing with a mental health issue	**Say the wrong thing**
“Not being able to properly answer a student’s question”	10. Answer a question incorrectly	**–**
	11. Be seen as the “expert”	*Not focus group tested*
	12. Overstep my boundaries	*Not focus group tested*
	13. See something as a small problem when really, it’s a big problem	*Not focus group tested*
	14. Be unable to help a student	*Not focus group tested*
	15. Be seen as judgmental	*Not focus group tested*
	16. Trigger an emotional reaction in myself	*Not focus group tested*

Note. Bolded text indicates wording changes that were made as a result of focus group testing

^a^Item recommended for removal due to similarity to Item 10

-- indicates no wording changes were suggested

### Validity and reliability

Assessing the psychometric properties of new instruments involves testing for both validity and reliability. Validity is described as the degree to which an instrument measures what it is intended to measure, and is determined by the “degree to which evidence and theory support the interpretations of test scores entailed by proposed users of tests” ([21] p. 9). Reliability refers to the consistency of test scores within a particular population. According to The Standards for Educational and Psychological Testing, validation of an instrument requires the accumulation of evidence from five sources: content; response processes; internal structure; relations to other variables; and test consequences. In this paper, we detail two types of validity evidence for the TCS-MH and WWMS, in addition to internal consistency reliability.

#### Content evidence

To gather content validity evidence, we used two methods. First, we conducted a focus group with eleven members of the Elementary Teachers’ Federation of Ontario (ETFO) (82% female, 18% male). Members of the ETFO were invited to volunteer to participate via an e-mail sent by a project team member. While traditionally, the recommended size for a focus group is 10–12 participants, with the ideal size being 6–8 participants [22], qualitative researchers recommend that that sample size be selected based on the researcher’s judgement, with consideration given to both the purpose of the research and the topic area in question. The goal of this focus group was to refine the item pools of the TCS-MH and WWMS as one component of the larger scale development project. Our sample was one gathered out of convenience, among of group of educators who were available to share their input regarding teaching about mental health in the classroom, and how confidence and worry may manifest in this context. We found our group of eleven participants to be sufficient for allowing idea sharing and varied perspectives on the topic, without providing so much data that it was unmanageable. Our purpose was not to reach data saturation, as is common with qualitative methodologies, but rather to gain insight from a select group of educational experts.

Prior to the session, the facilitator described the intention of each of the scales to enable participants to speak to the relevance of individual scale items. The scales were used as an interview guide, with participants reviewing each item in turn as a group, generating discussion and recommendations pertaining to item relevance and clarity. Where needed, detail-oriented probes were used to elicit further explanation from participants (i.e., “What do you mean by that?”, or “Can you tell me more about that?”) [23]. Note taking was conducted by a research assistant during the session. Notes were transcribed immediately following the interview to form a list of recommended changes to the scales. Suggested changes were reviewed by the authors, and revisions were made to each of the scales prior to moving forward to the next stage of research.

Next, we conducted a consensus survey modelled off of a traditional Delphi method with a wider panel of experts [24]. We used a sample of participants determined to be “educational experts” due to their post-secondary training in education and status as working teachers and/or educational specialists. Participants were recruited via e-mail invitation through the Education Faculty of an Ontario university. Systematic reviews of studies utilizing Delphi methods to investigate a number of topics have revealed sample sizes ranging from 5 to over 1000 expert panellists, with the majority ranging from 10 to 100 [25–27]. As the number of panellists increases, the probability of chance agreement decreases [28]. Our aim was to recruit a panel of at least 30 experts and we were successful in recruiting 33 (Table 3). This convenience sample was largely female (66.7%), with an average age of 43 (SD = 8.4) and an average of 15 years (SD = 6.2) of teaching experience. The majority of respondents reported that they themselves, or a close friend or family member lived with a mental illness (81.3%), while 100% of the respondents indicated having taught a student with a mental illness at some point during their teaching careers. Most respondents had never taken a course on teaching about mental health (65.6%), though over 70% reported having taught about mental health in their classroom on at least one occasion.

**Table 3:** Demographic Breakdown for Consensus Survey Sample (*N* = 33)

Variable	n	(%)	Mean (SD)
Sex			
Male	11	33.3	
Female	22	66.7	
Age			43 (8.4)
< 40 years	14	46.7	
≥ 40 years	16	53.3	
*Missing*	*3*		
Teaching experience			15 (6.2)
0–14 years	16	51.6	
15 years or more	15	48.4	
*Missing*	*2*		
Self or family/friend with a mental illness			
Yes	26	81.3	
No	5	15.6	
Unsure	1	3.1	
*Missing*	*1*		
Student with a mental illness			
Yes	33	100.0	
No	0	0.0	
Unsure	0	0.0	
Taken previous course on teaching about mental health			
Several times before	3	9.4	
Once before	8	25.0	
Never	21	65.6	
*Missing*	*1*		
Previously taught about mental health			
Yes	23	71.9	
No	9	28.1	
*Missing*	*1*		

Note. Valid percents reported in table (excluding missing data)

Missing data indicated where applicable

Participants were asked to rate the relevance of each item using a 3-point Likert response scale (1 = not at all relevant, 2 = somewhat relevant, and 3 = very relevant). We used these ratings to compute the content validity indices for each item (I-CVI), calculated by dividing the number of respondents who rated each item as “very relevant” or “very clear” by the total number of respondents [29]. Based on recommendations in the literature, items with relevance I-CVIs of 0.7 or greater were retained [29, 30]. Content validity indices for the scales in their entirety (S-CVIs) were calculated by taking the average of the I-CVIs for retained items only.

#### Internal structure evidence

In 2017–2018, a pilot test of the aforementioned teacher training guide was undertaken. The evaluation consisted of a one group pre-test, post-test design, with participants recruited through formal invitations sent to select Ontario school boards. Data were collected through an online survey presented to participants during registration for the teacher training guide. Recommendations regarding adequate sample size for factor analysis vary substantially. One guideline, for example, recommends that the number of subjects should be at least five times the number of variables [31]. Another suggests that a sample of 100 is suitable (Kline 1994), while others recommend samples in the range of 200–300 or more [32, 33]. Yet another source suggests that a smaller sample size is suitable, as long as factor scores are fairly strong [34]. Given the range of estimates in the literature, our aim was to recruit a sample size of 100 participants. We were successful in recruiting 93 (Table 4). This sample was largely female (77.4%), with an average (SD) age of 39 (8.3) and an average (SD) of 11 years of teaching experience (6.6). The majority of respondents reported that they themselves, or a close friend or family member lived with a mental illness (82.7%), while about 96% of the respondents indicated having taught a student with a mental illness at some point during their teaching careers. Most respondents had never taken a course on teaching about mental health (73.1%), though over three quarters (76.3%) reported having taught about mental health in their classroom on at least one occasion.

**Table 4:** Demographic Breakdown for Internal Structure Evidence Sample (*N* = 93)

Variable	N	%	Mean (SD)
Sex			
Male	21	22.6	
Female	72	77.4	
Age			39 (8.3)
< 40 years	51	54.8	
≥ 40 years	42	45.2	
Teaching experience			11 (6.6)
0–5 years	17	18.3	
6–10 years	33	35.5	
11–15 years	20	21.5	
More than 15 years	23	24.7	
Personal or family/friend with a mental illness			
Yes	77	82.7	
No	6	6.5	
Unsure	10	10.8	
Student with a mental illness			
Yes	89	95.6	
No	2	2.2	
Unsure	2	2.2	
Taken previous course on teaching about mental health			
Several times before	10	10.8	
Once before	15	16.1	
Never	68	73.1	
Previously taught about mental health			
Yes	71	76.3	
No	17	18.3	
Unsure	5	5.4	

We assessed the internal structure of the TCS-MH and WWMS using exploratory factor analysis (principal axis factoring). Retained factors were determined through the use of the Kaiser criterion, examining scree plots, and parallel analysis. Parallel analysis was conducted using R, Version 3.4.1. While this analysis was exploratory in nature, we hypothesized that a simple structure would emerge with all items loading on a single factor for each scale (as this had been our original intent in the scale development phase). Cronbach’s alpha was then calculated to estimate the theoretical internal consistency (reliability) of the test scores.

## Results

### Content evidence

Focus group participants considered twelve of the items on the TCS-MH to be overly long and complicated. Based on their recommendations, items were reworded using simpler language and were made more concise. Participants also made suggestions for reordering the items, as well as making the overall tone of the items more positive. Changes made to these items are shown in Table 1. Only two of the items on the WWMS were singled out for rewording. Changes made to these items are shown in Table 2.

Thirty-three educational experts participated in our online consensus survey. Table 5 summarizes the item content validity indices (I-CVIs) calculated for the relevance of each item. A total of 12 and 11 items demonstrated acceptable relevance I-CVIs (< 0.7) on the TCS-MH and WWMS, respectively, and were therefore retained for further analysis. The overall content validity indices for the scales in their entirety (S-CVIs) were .74 for the TCS-MH and .82 for the WWMS. Overall, these results provide support for the content validity of the TCS-MH and WWMS.

**Table 5:** Content Validity Indices for TCS-MH and WWMS (*N* = 33)

Tcs-MH content evidence		WWMS content evidence	
Items	Relevance I-CVI	Items	Relevance I-CVI
1. **I can answer my students’ general questions about mental health**	**.73**	a. **Trigger an emotional reaction in a student with a mental health difficulty**	**.81**
2. **I can create a mentally healthy classroom**	**.75**	b. **Cause a student to identify with a mental illness that they do not have**	**.73**
3. **I can advocate for the importance of learning about mental health**	**.76**	c. **Do more damage than good**	**.78**
4. I can spark interest in learning about mental health	.64	d. Cause students to second-guess their own mental health	.59
5. **I can help my students to be more aware of their mental health**	**.73**	e. **Glamorize mental illness**	**.70**
6. I can create engaging mental health lessons for my students	.67	f. **Single out a student who does have a mental health difficulty**	**.81**
7. **I can improve students’ general knowledge about mental health**	**.75**	g. **Say the wrong thing**	**.89**
8. I can ask my students engaging questions about mental health	.67	h. **Answer a question incorrectly**	**.93**
9. **I can help students to learn to value their mental health**	**.76**	i. **Be seen as the “expert”**	**.74**
10. **I can use students’ attitudes toward mental health to create learning opportunities**	**.72**	j. **Overstep my boundaries**	**.81**
11. **I can teach students how to find reliable information about mental health**	**.76**	k. **See something as a small problem when really it’s a big problem**	**.85**
12. I can create classroom activities that reinforce students’ knowledge of mental health	.64	l. **Be unable to help a student**	**.93**
13. **I can help to break down stereotypes about mental health**	**.78**	m. Be seen as judgmental	.67
14. **I can help students to learn about the negative impact of stigma**	**.73**	n. Trigger an emotional reaction in myself	.63
15. **I can improve students’ knowledge of resources available to support their mental health**	**.73**		
16. **I can improve students’ ability to seek help for mental health difficulties**	**.70**		
**Scale Content Validity Index (S-CVI)**	**.74**	**Scale Content Validity Index (S-CVI)**	**.82**

Note. Bolded text indicates item was retained for subsequent analyses and included in the calculation of the S-CVI

### Internal structure evidence

**Fig. 1:**
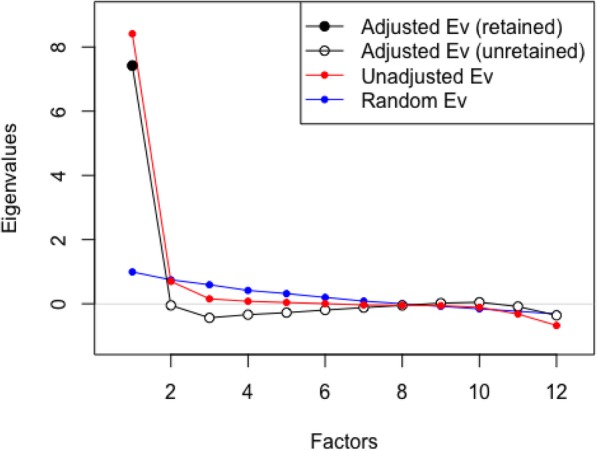
Parallel Analysis for TCS-MH

The Kaiser-Meyer-Olkin measure of sampling adequacy was 0.94 for the TCS-MH and 0.89 for the WWMS, with statistically significant Bartlett’s tests for sphericity (*p* < 0.001) indicating that items on both scales were suitable for exploratory factor analysis. Table 6 shows the factor loadings derived for each scale through principal axis factoring (PAF). As we hypothesized, A single factor solution was supported for both scales with all factor loadings above 0.65 by the Kaiser criterion, analysis of the scree plots, and parallel analysis. All items on the TCS-MH loaded strongly on a single factor with an unadjusted eigenvalue of 8.4 (adjusted 7.4) accounting for 70% of the variance in scores (Fig. 1 in Appendix).

**Table 6:** Factor Loadings for TCS-MH and WWMS (*n* = 93)

TCS-MH Item	Factor Loadings	WWMS Item	Factor Loadings
1. I can answer my students’ general questions about mental health	.70	1. Trigger an emotional reaction in a student with a mental health difficulty	.69
2. I can create a mentally healthy classroom	.71	2. Cause a student to identify with a mental illness that they do not have	.77
3. I can advocate for the importance of learning about mental health	.69	3. Do more damage than good	.80
4. I can help my students to be more aware of their mental health	.89	4. Glamorize mental illness	.71
5. I can improve students’ general knowledge about mental health	.87	5. Single out a student who does have a mental health difficulty	.72
6. I can help students to learn to value their mental health	.89	6. Say the wrong thing	.83
7. I can use students’ attitudes toward mental health to create learning opportunities	.91	7. Answer a question incorrectly	.77
8. I can teach students how to find reliable information about mental health	.81	8. Be seen as the “expert”	.66
9. I can help to break down stereotypes about mental health	.90	9. Overstep my boundaries	.84
10. I can help students to learn about the negative impact of stigma	.90	10. See something as a small problem when really it’s a big problem	.71
11. I can improve students’ knowledge of resources available to support their mental health	.88	11. Be unable to help a student	.68
12. I can improve students’ ability to seek help for mental health difficulties	.83		
**Coefficient alpha (α)**	**.96**	**Coefficient alpha (α)**	**.93**

Note. TCS-MH KMO Statistic = .94, Bartlett’s test for sphericity *p* < 0.001 (approx. Chi square 1233)

WWMS KMO Statistic = .89, Bartlett’s test for sphericity *p* < 0.001 (approx. Chi square 690)

**Fig. 2:**
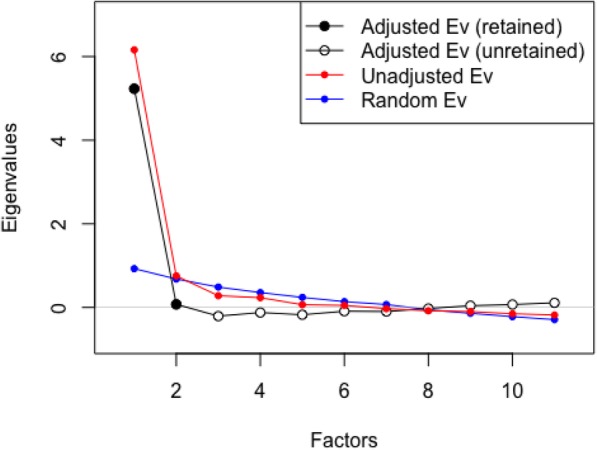
Parallel Analysis for WWMS

Though the parallel analysis recommended retaining two factors for the WWMS, we opted to use a single factor solution for this scale for two reasons. First, the second factor was very close to the cut off of zero (eigenvalue of 0.09), and secondly, a very drastic drop or “elbow” was evident in the scree plot following the first factor. Therefore, a single factor solution was chosen for the WWMS, with an unadjusted eigenvalue of 6.1 (adjusted 5.2) accounting for 55% of the variance in scores (Fig. 2 in Appendix). A Cronbach’s alpha of .96 for the TCS-MH scores and .93 for the WWMS estimated strong internal consistency (reliability) of the test scores. The Pearson’s *r* correlation coefficient between the scales was − 0.30 (*p* < 0.01), indicating separate, but related, constructs.

## Discussion

Teachers have an important role to play in creating mentally healthy, stigma-free classrooms and encouraging school-aged youth to recognize and modulate changes in their own mental health. Based on existing research regarding teachers’ self-efficacy, or confidence, we developed two new scales to fill a gap in the literature: the lack of domain-specific instruments for evaluating teachers’ feelings towards teaching about the topic of mental health. The TSC-MH was developed to assess teachers’ confidence in teaching mental health related topics to elementary school students. The What Worries Me Scale was developed to identify the issues that most worried teachers in presenting this content to their classes. The goal of this study was to report on the processes used to create these instruments and gather preliminary validation evidence.

We used a multi-stage development process that resulted in the collection of response processes, content, and internal structure validation evidence for these instruments. Content evidence was collected through the use of two methods. First, a focus group was facilitated with members of the Elementary Teachers’ Federation of Ontario who offered insight and recommendations regarding the clarity and interpretability of items on each scale. Secondly, an online consensus survey was conducted, modeled after a traditional Delphi method, where a group of educational experts rated the relevance of the individual items on each scale. Content validity indices were calculated using the relevancy ratings, and items with CVI’s over 0.7 were retained for subsequent analyses. Internal structure evidence was collected using the baseline data from a larger evaluation of an online training guide designed to provide teachers with the tools they need to deliver mental health-related lesson plans. As hypothesized, a single factor structure was supported for both scales, with each individual factor accounting for 70 and 55% of the overall variance in test scores on the TCS-MH and WWMS, respectively. Strong Cronbach’s alpha coefficients estimated strong internal consistency reliability of the scores. Also as hypothesized, the TCS-MH and WWMS were statistically significantly correlated, indicating separate, but related, constructs. Teachers who scored higher on the confidence scale had fewer worries about delivering the content, and vice versa. While these results provide promising preliminary evidence of validity, there are some limitations to this study.

Given that the scale development was situated in a larger evaluation project, there are several limitations to keep in mind. The sample size for the exploratory factor analysis was relatively small and overrepresented by female teachers between the ages of 30 and 39. Because teachers volunteered to be part of the larger evaluation, there is likely volunteer bias in the sample. We might expect those who volunteered to have a pre-existing interest or investment in creating a mentally healthy classroom and learning more about how to do so. Indeed, the majority of participants did report prior exposure to mental illness, among self, family, friends, or students, and had previous experience teaching mental health content to their students. This may account for the high Cronbach’s alpha values observed (over 0.9), which are higher than typically desired for a new instrument [35]. Future research is now needed to assess how these scales perform in larger, more heterogeneous samples of teachers. In particular, future work should consider examining the internal structure evidence for these scales using a larger sample size, particularly for the WWMS. Additionally, given the scale of this study which was situated within a larger program evaluation project, we were only able to collect certain types of evidence for validity. Therefore, this study presents only preliminary evidence of validation, and further testing of these scales is needed to investigate response processes evidence and relationships to similar and diverging constructs of interest (i.e., convergent and divergent validation). Finally, future work might also consider additional assessments of reliability, including test-retest reliability.

## Conclusion

We identified two unidimensional scales evaluating teachers’ confidence and worries regarding bringing conversations about mental health into the classroom setting. The Teacher Confidence Scale for Delivering Mental Health Content contains 12 items measuring educators’ confidence in their ability to positively influence student learning about mental health and mental illness; the What Worries Me Scale contains 11 items measuring the worries educators may have regarding the unpredictability of doing so, and the potential for negative outcomes. Using focus group testing, a consensus survey, and exploratory factor analysis, we collected preliminary validity and reliability evidence for these instruments.

To our knowledge, these are the first scales designed to specifically evaluate elementary school teachers’ confidence and worries associated with bringing conversations about mental health into the classroom. These scales may be useful in future evaluations of programs, educational workshops, or other initiatives designed to improve teachers’ overall confidence in teaching students mental health-related content. The WWMS, in particular, may be used as a jumping off point for schools looking to implement a training program of this kind, allowing program developers to pinpoint the areas most in need of attention among their teaching staff. It may be prudent for future research to investigate the utility of these scales among teachers at grade levels beyond Grade 7 and 8. While the research presented in this article does not address all aspects of validity, it does provide a preliminary analysis of evidence in support of the scales’ validity, and introduces two valuable domain-specific instruments to the literature regarding teachers’ self-efficacy that can now be used and further validated in subsequent research.

## Data Availability

The datasets used and/or analysed during the current study may be available from the corresponding author on reasonable request, and pending approval from Queen’s University Health Sciences and Affiliated Teaching Hospitals Research Ethics Board.

## Abbreviations

CVI: Content Validity Index
TCS-MH: Teacher Confidence Scale for Delivering Mental Health Content
TSES: Teachers’ Sense of Efficacy Scale
WWMS: What Worries Me Scale
